# The Hospital. Nursing Section

**Published:** 1902-12-20

**Authors:** 


// The Hospital.
**Nursing Section.**

Contributions for this Section of "The Hospital" should be addressed to the Editor, "The Hospital" Nursing Section, 28 & 29 Southampton Street, Strand, London, W.C.

No. 847.—Vol. XXXIII. *SATURDAY, DECEMBER* 20, 1902.

### Notes on News from the Nursing World.

### OUR CHRISTMAS DISTRIBUTION.

The large number of useful garments, upwards of 200, received this year from our readers were on view at The Hospital office from three to five on Tuesday last. Arranged on a long centre table and on smaller ones round the room, they made a goodly show, and many appreciative remarks were made by the visitors. Some nice little hoods for quite tiny babies, knitted, lined and quilted, with the woolly part inside, were greatly admired. There was quite a large number of babies' garments, among which were 18 pairs of knitted boots (white and pink and white and blue) from one contributor, and two "wee garments," beautifully made. One of our contributors is an old lady of 92, who sent in two excellent warm red flannel petticoats. "She is not well off," her nurse wrote, "but she helps all she can." Every gift in one parcel was accompanied by a pretty Christmas card. There is not space to enumerate the articles sent us, but they are all of a sensible and useful kind. The mounted shamrock sent from Nurse Ruel, of Ryde, was again much appreciated. The following have sent contributions since we last went to press :—Miss A. Burt, Lelant, Cornwall; Miss M. S. Healy, Downing House, Margate; Miss C. E. Brown, Woodbourne, Lastock, Bolton-le-Moors; "L. R.", Corbridge-on-Tyne; Miss Le Murehurst, Grosvenor Lodge, Queen's Road, Ryde; Miss M. A. Barnes Groom, 123 New Bond Street, W.; Miss Colhoun, Monks Hatch, Horsham; Miss J. Ratcliffe; the Editors of The Hospital and Mrs. Binnie; Miss E. Kirkley, Dowsing Institute, Bournemouth; Miss Clarke. The list of hospitals which are to receive the garments will be published next week.

### THE ROYAL RED CROSS.

The King has been graciously pleased to confer the decoration of the Royal Red Cross on Miss Annie J. Weighell, superintendent at the Countess Roberts's Officers' Hospital and Nurses' Home, Murree, India. Miss Weighell was trained at the Royal Infirmary, Bristol, and has been nursing in India for 12 years. The decoration has also been bestowed by the King on Miss Ker Dunlop for her services in connection with the care of sick and wounded soldiers during the recent war in South Africa.

### THE WOMEN'S MEMORIAL TO QUEEN VICTORIA.

We are able to state that the money collected for the Women's Memorial to Queen Victoria will probably be presented, early in the ensuing year, by the Marchioness of Londonderry, the president, to the King and Queen, who, subsequently, will hand on the amount to the Queen Victoria Jubilee Institute for Nurses. It is not yet decided whether there will be any public ceremony. With regard to the sum received so far, we understand that at present it is about £60,000 from nearly three million contributors. The executive committee are anxious that during the next five or six weeks it should be increased to £70,000, and equal the fine record of 1887. We trust that as some far corners of the Kingdom have not yet responded to the appeal, the additional £10,000 will be obtained.

### PRINCESS LOUISE AND QUEEN'S NURSES IN SCOTLAND.

Last week the Princess Louise, Duchess of Argyll, visited the Scottish district home of the Queen Victoria Jubilee Institute for Nurses in Castle Terrace, Edinburgh, and was received by Lord Kinross of Glasclune, who is one of the vice-presidents. Previous to her inspection of the home the Princess presented badges and certificates to a number of Queen's nurses. These included Miss Myers, of Edinburgh, and Miss Farrell, of Glasgow, who received silver badges on their appointment as superintendents. Twenty-one nurses who have qualified as Queen's nurses also received badges, and twelve who have completed two years' engagement with the Institute after their training, certificates.

### NURSING THE BISHOP OF LONDON.

The Bishop of London is not, we are glad to hear, seriously ill. But the attack of influenza from which he has been suffering has made him very weak, and he is being nursed by Miss Haines, one of the nurses who attended the King in his late illness.

### ROYAL NATIONAL PENSION FUND AND PREMIUMS IN ARREAR.

As the third quinquennial period of the Royal National Pension Fund for Nurses closes on December 31st, it will greatly facilitate the work of the Fund if all premiums under policies are forthwith paid up to the end of the year, in accordance with the terms of the circular letter sent out by the secretary on April 30th last. We have no doubt that this reminder will have the desired effect.

### THE NEW MATRON OF CHELSEA HOSPITAL FOR WOMEN.

We announce with pleasure that Miss E. M. Edwards has been appointed matron of Chelsea Hospital for Women in succession to Miss Heather Bigg, who was lately elected matron of Charing Cross Hospital. Like Miss Heather Bigg, Miss Edwards returns to an institution where she is already well known and esteemed. After her training at St. Bartholomew's Hospital, she was for more than a year night sister and assistant matron at the Chelsea Hospital for Women. During the interval of three years, she has been matron of the Ilkley Hospital and Convalescent Home, a position which she has filled with signal ability and energy. Miss Edwards will be warmly welcomed back to London as the head of a special training school of considerable importance.

### THE TRAGIC DEATH OF A NURSE AT THE LONDON HOSPITAL.

It is not possible to exaggerate the sorrow caused in the London Hospital by the shocking fate of Miss Margaret Douglas, one of the nursing staff, who was burnt to death a few days ago. Unfortunately, Miss Douglas had acquired a dangerous habit of leaning against the mantelpiece with her back towards the fire when she was talking. She had been repeatedly warned of the peril she incurred, but apparently without avail. At ten o'clock on the morning of Sunday, December 7th, she was in the kitchen adjoining the diphtheria ward, chatting with a sister. As she came into the ward, Miss Marian Thomas, the sister-in-charge, noticed that smoke was coming from her skirt at the back. Suddenly it flamed up, and Miss Thomas exclaimed, "Stand still while I put it out." But Miss Douglas was so beside herself with fright that she rushed right up to a cot where a child was in bed and threw herself upon it. The sister, with much quiet courage and presence of mind, succeeded in dragging her away before the fire had spread to the child and its surroundings. She then seized a blanket and tried to wrap it round the burning nurse, but the latter broke away again and ran towards the door. Ultimately, with the assistance of another nurse, Miss Douglas was got on to the floor and the flames were extinguished with a rug. Every care was given to the poor girl, but she died on the Tuesday evening following, and at the necessary inquest a verdict of "accidental death" was returned. The mother of the deceased lives in Wiltshire, and when the matron found that it was impossible to send a telegram immediately after the occurrence, owing to the accident happening on Sunday, she at once dispatched a nurse to break the terrible news and bring Mrs. Douglas back to the hospital as soon as possible. They caught the return Sunday train, and, thanks to the kind consideration of Miss Lückes, the distressed mother had the melancholy satisfaction of being with her daughter until she passed away.

### A NURSING PRIZE.

The nurses past and present of Sir Patrick Dun's Hospital, Dublin, have given expression to the affectionate regard and sincere admiration in which they hold Miss Margaret Huxley, who is relinquishing her post as matron of the hospital after eighteen years of continuous work. They have subscribed a considerable sum of money and handed it over to the governors of the hospital, in trust, for the purpose of founding a gold medal to be called "The Margaret Huxley Prize." This prize is to be given biennially to the best nurse trained in the hospital, the decision being left in the hands of the matron for the time being. Good conduct and general nursing ability, together with the marks obtained in the theoretical examinations, are to be taken into account. This is both a unique testimonial, founded by nurses for nurses, and also a lasting memorial of the appreciation in which Miss Huxley was held by those for whom she worked and amongst whom she laboured, whose interests she has always sought to advance. It is interesting and pleasing that this spontaneous tribute comes from Irish nurses to their English matron, whom they now look upon as one of themselves.

### PORTSMOUTH PARISH INFIRMARY.

The probationers of Portsmouth Parish Infirmary have been preparing their Christmas entertainment for the patients for some weeks past. On December 27th and 31st the "Flower Masked Concert Troupe" will give a performance, including singing in character and otherwise, and mandoline selections. Among the troupe are to be Snowdrop, Honeysuckle, Ivy, Violet, Lily, Tulip, the accompanists being Sisters Mistletoe and Holly. The concert is to be followed by the farce, "A Perfect Cure," in which four nurses will act.

### CONCERT AT GUY'S HOSPITAL.

The concert at Guy's Hospital, given by the Nurses' Choral Society and Students' Glee Club last week, was a very successful one. Part songs were sung by the combined choirs, and the nurses gave a trio, entitled "Snow;" Sister Rennie sang "Tears," very sweetly, and Sister Patience gave "A Summer Night." Part II. consisted of a cantata, "The Banner of St. George," with a solo by Nurse Pettit. A large number of nurses and students and their friends were present. The proceeds of the sale of tickets will be devoted to the Nurses' League.

### EDINBURGH ROYAL INFIRMARY AND ROMAN CATHOLIC NURSES.

The Edinburgh papers are so inundated with letters from correspondents on the subject of the employment of Roman Catholic nurses in the Edinburgh Royal Infirmary, that one of our contemporaries declines to publish any communications that are not signed by the writers. We have read some of the letters which are signed, and one of them at any rate, is only a tirade against the Roman Catholic religion. The single practical question at issue is whether nurses are excluded from the Edinburgh Royal Infirmary because they are Romanists. The matron says that there is no rule on the subject, but she also states that in the course of several years she has had responsible relations with several hundreds of nurses, only one of whom was a Roman Catholic. This statement is construed by Father Power as an admission of his charge that "no Catholic need apply," and as justifying his criticism of her methods in a sermon which he preached on behalf of the infirmary. But there may have been reasons, apart from religion, why the nurses engaged by Miss Spencer have nearly always been Protestants. Father Power affirms that there have been Romanist nurses at the infirmary who concealed their religion in order to secure and to retain their appointments; and while "no advocate of moral cowardice," he rather tries to find excuses for these. As, however, there are plenty of hospitals in Scotland, as well as south of the Tweed, at which no attempt is made, either directly or indirectly, to impose a religious test, there is no occasion for moral cowardice. We should blame Miss Spencer if, going beyond the printed questions she is allowed to put to the candidates, she intrudes on the private domain of conscience; but in the absence of direct evidence that she has done so, we must conclude that she has loyally adhered to the spirit of the regulations for the admission of probationers.

### THE MATRON OF FOLKESTONE SANATORIUM.

The grounds of the resignation by Miss L. A. M. Hughes of the post of matron of the Folkestone Sanatorium, were mentioned in a former issue. That the action of Miss Hughes, which was followed by that of the nursing staff, is regretted in Folkestone, is proved by the testimonial which has been presented to her by members of the medical profession in the town. In the address accompanying the handsome gold watch and chain which have been given her, the signatories, 20 in number, record their appreciation of the excellent manner in which she has performed her duties as matron of the Hospital for Infectious Diseases during the last three years. They add: "Your unselfish devotion to the patients, your tact, kindness, and unflagging energy have gained you our highest esteem. In cases of emergency you have shown knowledge and resource. The nurses under you have been well trained and efficiently supervised by you. It is with sincere regret that we hear of your resignation."

### CHRISTMAS PREPARATIONS AT LONDON HOSPITALS.

Christmas will be celebrated at most of the hospitals in the Metropolis with the usual festivities and entertainments. At the London Hospital, the sisters, nurses and probationers will go through the wards early on Christmas morning, singing carols; there will be a visit from Father Christmas later in the day, a Christmas dinner of roast beef and plum pudding, and subsequently, festive ward teas and a series of entertainments by doctors, students and friends, the men being allowed to smoke. As Christmas Day is on Thursday, the entertainments at King's College Hospital, which usually take place the day following Boxing Day, will be postponed to Monday, the 29th. There will be a series of concerts on that day in the wards, beginning at half-past three and ending about 7.30. Presents will be given to the patients on Christmas Day, and the dinner will consist of roast turkey, plum pudding, etc. The wards of the National Hospital for Epilepsy and Paralysis will be decorated with plants and flowers, and on Christmas Day there will be as many ward teas as possible. Later on, there will be a series of entertainments in the large hall, the dates of which will be fixed shortly. It is expected that about two-thirds of the patients will be able to attend. There will also be a tree for the children, of whom there are about twenty in the hospital. As there is no large room at the Homœopathic Hospital, the entertainments will take place in the wards, and will be very simple in character. The nurses will sing carols, and the children will have a tree lighted with electric light. Presents of warm clothing will be given on Christmas Day.

### SWANSEA HOSPITAL AND PRIVATE NURSING.

There has been a misunderstanding about the change which has recently been initiated at the Swansea General and Eye Hospital. A private nursing staff on a very small scale was first attached to the institution nine years ago, and it was always intended to develop the movement when the new building for additional accommodation of nurses became available. There has been a difference of opinion among the members of the board as to the policy of this step, but nothing could be more unfair than to suggest in the face of ascertainable facts that it is intended to make money for the hospital out of the earnings of the private nurses. In the report of the hon. medical staff of the Swansea Hospital for 1901-2, which was drawn up by Dr. Brock and adopted by his colleagues, there is the following passage: "We consider that a hospital should be quite content that the private nursing staff should pay its own expenses, and that the nurses should have their own earnings." So long as this principle is adhered to, and assuming that the sick poor in the hospital will continue to receive the same attention they have hitherto enjoyed, there can be no cause for complaint.

### BARRISTER AND NURSE.

At Leeds Assizes last week an action for breach of promise of marriage was brought by Miss Selina Neesham, who appeared in court wearing the uniform of a nurse, against Mr. William Kendall, a barrister and landowner who lives with his mother at the plaintiff's native village. The report of the proceedings in the daily papers is headed "Barrister and Nurse," and Miss Neesham was awarded £550 damages, to which she was doubtless fully entitled. As, however, she stated that she was under twenty, we question her title to wear the uniform of a nurse. At any rate, she cannot have been fully trained.

### THE FIRST YEAR AT SOUTH BRENT.

The first annual meeting of the South Brent District Nursing Association has just been held. The district nurse paid 1,516 visits during the year, and the report also shows that the receipts for the period amounted to £127, of which £45 was obtained as the result of a bazaar held in August, and a lecture and a concert yielded between £11 and £12. The Association is happily in the position of starting another year with £40 18s. in hand.

### SHORT ITEMS.

At a meeting of the Central Midwives' Board held last week at the Privy Council Office, all the members being present, Dr. Champneys was unanimously appointed chairman, and it was resolved to apply to the Privy Council for permission to appoint a secretary.—The services of Miss Ethel Procter have been accepted by the Church Missionary Society, and she goes to Persia to nurse under their auspices. Miss Procter is a trained nurse, and has been working in Newcastle-upon-Tyne. Nurses will be interested to learn that the special prize of two guineas offered by the proprietors of the *Academy and Literature* for the best tale suitable for reading aloud to a child, was awarded to Mr. Charles Cutting, of the Royal Free Hospital, Gray's Inn Road, an occasional contributor to The Hospital.

### Lectures to Nurses on Clinical Chemistry.

By Carstairs Douglas, M.D., B.Sc., F.R.S.Edin., Professor of Medical Jurisprudence and Hygiene, Anderson's College Medical School, and Dispensary Physician, Samaritan Hospital, Glasgow.

### LECTURE II.

In our first lecture we made a little excursion into the domain of theoretical chemistry in order that certain terms might be made clear to you; and this done, we need not repeat it. It was pointed out that the acidity of urine was due not to an acid itself, but to an acid salt, the precise salt being acid phosphate of soda. I should like to give you a little hint here about taking the reaction. Sometimes when the litmus paper is dipped in, it is almost impossible to say whether it is really altered in colour or merely darkened by being wet. This difficulty can be overcome by the simple plan of wetting the strip of paper in ordinary water before dipping it in the urine. It will then be easy, as a rule, to say whether the tint is further altered or not.

In health the urine may become alkaline where a great deal of vegetable food is taken, owing to alkaline salt derived from such food being excreted in the urine. Even after an ordinary meal it may become alkaline, because the secretion of the acid gastric juice renders the blood (and so the urine) more alkaline. Litmus paper in such cases changes from red to blue, and if dried retains its colour because the alkali present (usually soda) is a fixed one, not driven off by warmth. But many of you must have noticed that urines occasionally have a very disagreeable and pungent smell like ammonia, especially in cases of bladder inflammation, and where a sample has been kept too long in a warm room. Such urines are said to have undergone "ammoniacal decomposition," and the ammonia comes from urea, the most important solid in urine, which, when urine is allowed to stagnate where micro-organisms can get at it, ferments and produces this body. Ammonia is also an alkali, and turns red litmus blue; but if the test-paper be dried with gentle heat, the blue colour goes, and so we term this alkali "volatile."

### Specific Gravity of Urine.

This need not detain us long. It is ascertained by allowing the urinometer to float in a test-glass of urine, and noting what figure on the scale is just cut by the level of the fluid. Foreign substances merely floating in the urine do not affect the specific gravity; it is only altered by what is dissolved in the liquid. In health the density of urine varies from 1015 to 1025, pure water being taken as 1000. If the figure obtained is very low, *e g.* 1005, one may suspect waxy disease of the kidney, chronic interstitial nephritis (cirrhotic and contracted kidney), or diabetes insipidus. If raised much, *e.g.* to 1035 or 1040, the urine may prove to be that from a case of diabetes mellitus, it being the dissolved sugar and urea that raise the density. If you wish to take the specific gravity and find you have only half a glassful of urine, so that the urinometer will not float, what are you to do? You may add an equal volume of water, mix well, and then ascertain the density, thereafter multiplying the last two figures given by the urinometer by two. For example: if the diluted urine give 1012, the real specific gravity of the specimen will be 1024.

### Colour of Urine.

This is generally described by some well-known phrase such as straw-coloured, amber, and so forth. The urine may be of a much deeper hue, especially if concentrated. The most important changes in colour caused by disease are those due to blood and to bile. When a patient passes urine containing blood, he is said to suffer from "hæmaturia," and the urine is then, in typical cases, of a dull brownish-red; such urine is called "smoky." The fresher the blood, and the lower its source in the urinary tract, the redder will the urine appear.

When a person has bile in the urine, he exhibits "choluria," and the urine is then of an olive green, or more frequently brown, tint with a suggestion of green in it. If doubt exist, cover the glass with your hand and shake well; the froth in bilious urine is yellow. Sometimes the urine of persons who have taken carbolic acid is of a greenish tint simulating that in choluria, but the froth is white. Santonin, a drug often given for worms, may make the urine bright yellow, while after rhubarb and senna it may appear of an orange tint.

### Quantity of Urine.

A healthy adult passes 40 to 50 ounces per diem. The amount is lessened by free perspiration, vomiting and diarrhœa, and increased by drinking abundantly. In hysteria large quantities of pale limpid urine are often passed. The amount is also increased in waxy disease of the kidney, chronic interstitial nephritis, and diabetes insipidus and mellitus. Such cases are said to exhibit *polyuria* (meaning "much urine"). In contrast to this we have the condition of *oliguria* ("little urine") where the flow is lessened. This is met with in acute and ordinary chronic Bright's disease; in cases where there is great perspiration, diarrhœa, and violent vomiting; and in poisoning by cantharides, arsenic, and turpentine. If the urine is totally suppressed, the term *anuria* ("no urine") is employed. Drugs which increase the secretion are called *diuretics.* As examples we may quote digitalis, alkaline salts of potash, squills, and diuretin.

### Sediments in Urine.

It is of great advantage for a nurse to be able to know the nature and the significance of naked-eye deposits in urine, and, indeed, in many hospitals such knowledge is indispensable if the examinations for a diploma are to be passed successfully. There are only some half dozen deposits which are very important, and these we will consider briefly.

1. *Urates.*—These are salts of uric acid, and always exist in urine. Through some change in that fluid they may be precipitated or thrown down in an insoluble form, and so produce a deposit. In their fall they carry down with them colouring matters in the urine, and so the deposit often appears red, reddish-yellow, or pink. It may be white, however. The urine above is often slightly turbid, and the sediment is often described as "brick-dust," a very good name. The urine in such cases is always *acid.* Urates do not deposit in an alkaline fluid, and therefore the addition of a little caustic potash (liquor potassæ) to the urine will make the sediment disappear. Further, such a deposit is dissolved by heat, and therefore if a little be gently warmed in a test-tube it will clear. From this it is obvious that the sediment of urates is not found in freshly-passed (warm) urine, but only becomes apparent as the latter cools. Remember then that such urine is always acid, and that the addition of an alkali, or the application of heat, dissolves the precipitate.

2. *Uric acid.*—Where the acid is deposited in a free state, *i.e.*, not combined with a base, the deposit is scanty, reddish in tint, and appears as small, separate, crystalline granules. It is called the "cayenne-pepper" deposit. The urine is always acid; alkalies dissolve the deposit, but it does not disappear when heat is applied.

### The Report of the Departmental Committee on Workhouse Nursing.

In considering the report of the Departmental Committee on Workhouse Nursing the recommendations therein contained may be regarded from several points of view, and so no doubt they will be regarded by the various groups of persons interested. As is only proper we may first consider how they are likely to affect the sick poor themselves; then comes the question how far they will relieve the friction which the nursing problem has introduced into workhouse infirmary administration; then we have to consider the nurses themselves in their various grades; then so important a series of recommendations as these, involving as they do a sort of Government recognition of certain standards of training, must have a notable influence upon the status and the prospects of a large number of sick nurses who personally have no direct connection with poor-law nursing; and finally the public at large are likely to have their own point of view which also must be borne in mind. Hence it is that our opinion of this very careful and painstaking report cannot be expressed in any single phrase or even in any short sentence.

### The Sick Inmates.

As to the patients, when one considers the recommendations that the employment of untrained "assistant nurses," as they have been called, shall be discontinued; that the nursing of the sick shall be entirely in the hands of those who either have already gone through a course of training or are still, as probationers, undergoing such a course, and are therefore presumably young, active, intelligent, and (even as a matter of mere self-interest) full of enthusiasm in their work; that the "stores, food, bedding, and other articles for the use of the sick wards," instead of being doled out by the matron, shall be "requisitioned" by the trained superintendent nurse; and finally, so far as concerns small workhouses, that "at least one trained nurse, preferably qualified in midwifery should, if possible, be resident in every workhouse," and that "in very small workhouses where it is not feasible to have a resident trained nurse, arrangements should be made for procuring at short notice the services of a temporary trained nurse"; when we consider all these things we cannot fail to see that in many workhouses the recommendation will act greatly to the benefit of the sick inmates.

### The Administrative Question.

There seems every probability that the recommendations made in the report will go far to lessen the friction which has at times occurred in regard to the nursing in workhouse infirmaries. This friction has arisen to a large extent from the fact that the position of the superintendent nurse has been ill defined. As the report says "in position she was on a level with the master and matron, in so far as the Guardians could not dismiss her without the Board's consent . . . yet at the same time the exercise of her authority was to a large and not altogether certain extent 'subject to the directions' of the master or matron." The report very truly says that "in such a situation any master or matron, who resented the creation of the new office, could clearly find ample opportunities for increasing the difficulties which the new officer would naturally encounter in settling down into her position." On the other hand cases appear to have occurred in which a nurse has seemed to be "more impressed with the necessity of emphasising the dignity, rather than the utility, of her office." Indeed, cases have occurred in which superintendent nurses, holding to the strict letter of the Order under which they were appointed, have "successfully insisted on their right not to be compelled to perform actual nursing duties in connection with the sick." They were "superintendent nurses" and they declined to do anything but superintend, and apparently they were within their rights. Then there is the constant warfare in regard to the matron. Many of the duties of the nurses still remain, according to certain clauses which have not been repealed, the duties of the matron, duties which she is not only allowed, but is bound, to perform. Hence constant friction in regard even to purely nursing questions. The master also has a multitude of duties in regard to the sick which he can by no possibility perform, while "in contrast to this incessant and ubiquitous responsibility for and control of the sick, which technically vest in the Master and Matron, the Superintendent Nurse has no specific authority or duties at all in connection with the sick, except such as devolve upon her indirectly." The wonder is that under such circumstances things have gone on as well as they have done.

According to the recommendations in the report, however, all this will be altered. The master remains master of the establishment. This is definitely laid down. He goes nowhere on mere sufferance; he is to retain "his full authority and responsibility as Officer in charge of the Workhouse;" the "right, but not the duty," of entry into the sick wards is to be maintained; and when the Master traverses the sick wards and "at all other times" the power to report any neglect of duty to the Visiting Committee is to be "distinctly recognised as within his rights." But beyond this the governance and management of the wards is to be practically left in the hands of the superintendent nurse, who will have full control not only of the nurses but of the servants and pauper helpers who may be engaged so long as they are in the wards. As to the matron, except when acting for the master, she will have but little to do directly with the nurses beyond being responsible to the Guardians for the cooking and for the washing and mending of the clothes. Certainly, so soon as the first rub has been got over the recommendations will tend to harmony by removing points of contention.

### The Nurses and Probationers.

Whatever tends to diminish friction in the administration tends to improve the position of the nurses and to add to the attractiveness of the service. But beyond the advantages so gained many of the recommendations in the report go directly to increase the comfort of the nurses. Practically the abolition of the assistant nurses is likely to be a great gain, not merely by giving occasion for a larger number of appointments, but more especially by ensuring to the superintendents and other nurses that the carrying out of their directions will not be left to unskilled hands, and by ensuring to the probationers a more congenial companionship in their work than is sometimes the case at present. The granting of a preliminary trial of six or eight weeks before being finally appointed as a probationer will be of great advantage not only to those entering the service but also to the superintendent nurse, seeing that it will give to her "what may legitimately (it is submitted) be conceded to her, viz., a voice in the selection of the probationers." Then several distinct ameliorations in the nurses' lot are proposed, such as the employment of paid servants to look after the nurses' rooms, more comfortable quarters, separate bedrooms, increased opportunities for promotion to the office of matron, the granting of a higher salary in country than in town workhouses to compensate in some degree for the greater monotony of the work, an increase in the amount of "leave" and its distribution on a definite scale (three weeks

### THE REPORT OF THE DEPARTMENTAL COMMITTEE ON WORKHOUSE NURSING—*Continued.*

a year, one day a month, alternate Sundays, half-day a week, two hours a day, which, although less than at some hospitals is far more than obtains at present in many workhouses), the granting to nurses of some choice in the character of their "rations," the encouragement to guardians to pay part of the premiums towards annuities for their nurses. To those entering the service there would seem to be very definite advantages in the recommendations made as to the training of probationers. Although it is proposed to abolish the untrained assistant nurses it is by no means suggested that the whole nursing of the sick in workhouse infirmaries should be given to fully-trained—*i.e.* three years' trained—nurses. By way of increasing the number of probationers, and utilising some of the smaller infirmaries as training schools, it is proposed to establish two grades of training schools and two grades of nurses. The "Major Training Schools" will, as at present, give a three years' course, which will give right to the title of "trained nurse" and fit her for the acceptance of any post in the service except that of superintendent nurse for which post a year's service as a trained nurse after obtaining her certificate will be required, in addition to a certificate of midwifery recognised by the Board of Midwives. The "Minor Training Schools" will give a one year's course, granting the title of "qualified nurse," which will enable her to fill any position where the supervision of a trained nurse is available. Moreover, there is a provision by which a nurse entering at a minor school is to be enabled, should she take to nursing seriously, to go in for the higher certificate at a major training school without doing the whole of the course over again. There can hardly be a doubt that all this will be a popular arrangement, and will draw many probationers; the offer of a one year's training followed by a government certificate of "qualification" (!) and freedom to go out into the world and compete with "three-years' trained" nurses for private work will, as the committee evidently intend it shall, draw into the nursing service a considerable number of young women who, as the committee describe it, "enter merely for the acquiring an excellent training as a nurse," all of which may be very well as a means of fishing for probationers, but may, as we shall show later, be far from well for the nursing profession at large.

(*To be continued.*)

### A SUMMARY OF THE RECOMMENDATIONS OF THE COMMITTEE.

For the convenience of our readers we append a summary of the recommendations of the Departmental Committee:—

### The Probationers.

With regard to the qualification for probationers, they must be at least 21 years old, have good character and health, possess intelligence, and, if possible, a preliminary trial period of at least six weeks or two months passed in the sick wards to the satisfaction of the guardians. An increase in the supply should be fostered by increasing the facilities for training; by increasing the opportunities for occupation and promotion in the Poor Law service when the training is complete; to which end the appointment of matrons of workhouses who have received training as nurses should be encouraged. All probationers should be enabled to qualify, if they desire, as trained nurses and superintendent nurses.

As to training, probationers should in future be permitted to train in two classes of schools—minor and major—and the board's sanction should no longer be required to individual appointments as probationers. No more appointments as assistant nurses should be sanctioned.

### The Nurses.

The board should in future recognise two classes of nurses, viz. qualified nurses holding the certificate of a minor training school or an equivalent certificate from a non-Poor Law institution, eligible for any appointment where the supervision of a trained nurse is available; trained nurses holding the certificate of a major training school or an equivalent certificate from a non-Poor Law institution, eligible for any appointment other than that of superintendent nurse. It is also suggested that the supply should be increased by augmenting the number of probationers and increasing the attractiveness of Poor Law nursing. That in order to ensure that there shall be a proper distribution of the supply of nurses among the various workhouses the Committee very strongly recommend that the adhesion of boards of guardians to the following principles should be obtained:—

At least one trained nurse, preferably qualified in midwifery, should, if possible, be resident in every workhouse; but where the ordinary number of occupied sick-beds does not exceed 60, the matron of the workhouse may, if she is a trained nurse, act as such, with such suitable assistance in the shape of an assistant matron and of trained or qualified nurses as may be required.

In very small workhouses where it is not feasible to have a resident trained nurse, arrangements should be made for procuring at short notice the services of a temporary trained nurse upon an emergency.

That the responsibility for providing an adequate staff of both trained and qualified nurses in each workhouse and infirmary rests primarily upon the guardians, acting with the advice of their medical officer; that the attention of guardians should be called to their power to prescribe appropriate duties for their nurses.

### The Superintendent Nurses.

A superintendent nurse should be "a trained nurse" with the additional qualifications of at least one year's service as a trained nurse, and a midwifery certificate recognised by the Board of Midwives. It is also proposed to transfer to the superintendent nurse the duties of the master and matron as to the reception, care, and control of the sick and convalescents in the sick wards; the control of the nurses; the control of the paid and pauper servants while in the sick wards; the visiting of the sick wards; the cleanliness of the sick wards and the furniture and fittings in them; the care and distribution of clothes, bedding, and of all stores in the sick wards; and to the reception, service, and distribution of food in the sick wards.

In addition, the following duties should be given to the superintendent nurse:—To obey such rules laid down for her by the guardians as may be reasonable and conformable to her office; to report in writing to the guardians; to assist in the actual work of nursing patients and otherwise to perform the ordinary duties of a nurse on an emergency, or if so required by the guardians; to summon the master or matron to the sick wards upon request of a patient or upon any emergency involving the safety of the sick wards or their inmates, and to consult and report to the master in any difficulty; to attend, on the repuest of the master or matron, in the receiving ward upon the admission of a pauper deemed to be ill, and to advise as to moving such pauper to the sick wards in the absence of the medical officer, and to provide in like manner for the nursing of any sick pauper unable to be moved to the sick wards; to requisition from the master of all stores, food, bedding, and other articles for the use of the sick wards, and to be responsible for the same when delivered into the sick wards; to report to the master, either when the necessity arises, or when a desire is expressed by any patient for the presence in the sick wards of any person or persons, including ministers of religion; to report to the master the name of any inmate under her charge desirous of making a complaint or application to the guardians; to report to the master the occurrence of any death or birth in the wards under her charge, together with all particulars necessary for the registration of the same; to report to the master any structural defects in the sick wards.

THE MATRONS.

If properly trained, the matron should be allowed a trained nurse in a workhouse with not more than 60 sick beds; a superintendent nurse in a workhouse with not more than 100 sick beds. In each case she should be assisted by a proper staff of nurses, and by an assistant matron when necessary.

THE GUARDIANS.

Power should be given to the guardians to appoint individual probationers without the board's sanction; to prescribe appropriate duties for a superintendent nurse; and they should be urged, when possible, to separate sick wards from main building of workhouse.

GENERAL.

That the basis of the grant to guardians under the Local Government Act, 1885, should be revised so as to enable the State to contribute more directly to the cost of the Poor Law nursing service; and that the grant, so far as nurses are concerned, should only be paid in respect of nurses whose qualifications and appointments are in accordance with the Local Government Board's requirements.

### Presentations.

SOUTHAMPTON INCORPORATION INFIRMARY, SHIRLEY WARREN.—Dr. J. Hindshaw, resident medical officer of the Southampton Incorporation Infirmary, on leaving was presented with a brown morocco dressing case from the nursing staff, as a mark of esteem and with best wishes for his success in future.

THE HOSPITAL, KENDAL.—Miss Emily Mann, who is leaving Kendal Hospital to become sister of the children's ward at West Ham Hospital, has been presented by the matron, nurses, and patients with an address, an afternoon tea-service, oak tray, silver teaspoons, and sugar tongs.

### Novelties for Nurses.

BY OUR SHOPPING CORRESPONDENT.

GOLF IN THE HOME.

THERE are very few means by which nurses can utilise short periods of leisure by enjoying healthful exercise and recreation. Yet most hospitals and kindred institutions possess large vacant spaces, covered or uncovered, which are well adapted for recreation under suitable conditions. These conditions are met by the system of "Home Golf," which has been arranged by Messrs. Anderson and Anderson, the well-known manufacturers of indiarubber goods, at 37 Queen Victoria Street. The Home Golfer is an arrangement whereby a captive ball can be hit with the full force and precision which would be brought into use on the golf links, and the length and elevation of the drive are both recorded by a mechanical method. The Home Golfer affords interesting and admirable practice.

### Victorian Trained Nurses' Association.

BY THE HONORARY SECRETARY.

THE first annual meeting of the Victorian Trained Nurses Association was held in the Athenæum, Collins Street, on Monday, October 13th. Lady Clarke, Lady Madden, Miss Madden, Mrs. I. A. Levy, the President of the Melbourne Hospital, and others interested in nursing were present. The chair was taken by Mr. I. A. Levey, the Vice-President.

The hall was well filled with nurses wearing indoor uniform, who took a keen interest in the proceedings. The report and balance-sheet having been read and adopted, addresses were given by different members of the Council.

A motion was proposed by Dr. Duncan, of Kyneton, that the country hospitals should have representatives on the Council. This was carried, the number being limited to six, two members for each of the three sub-centres.

The year's work has been very satisfactory. Nearly 700 nurses have been registered: 90 of that number were admitted under Clause 21, which reads as follows, and which, it will be noted, expired last June:—

"21. Any nurse also who can show to the satisfaction of the Council that she has been engaged for not less than three years in the *bona fide* work of medical and surgical or special nursing, either in hospitals or in private nursing, and can produce certificates of competency and good conduct, satisfactory to the Council, from at least three reputable medical practitioners as to her attainments and repute shall be eligible for registration up to June 30th, 1902, subject to the approval of the Council. Provided always that the Council shall have power in any case to direct that any candidate under this rule may be examined in practical nursing by three examiners appointed by the Council, and provided, further, that in the case of nurses registered under this rule the qualification in the register shall stand as 'Admitted by the Council under the provisions of Rule 21.'"

A great many of those 90 have been trained in private hospitals, and some are really good nurses, though their training has not been in recognised hospitals.

All the country hospitals have agreed to accept the curriculum drawn up for the Victorian Nurses' Association, and to either send their trainees to Melbourne for their final examination or to one of the sub-centres appointed for that purpose. So that in future every nurse must have trained for three years, and must have passed the examination held by the Conjoint Board of Examiners, before she can be registered. No nurse will be accepted in a special hospital for training in special work who has not previously been registered as a general nurse. The homes for private nurses are most of them registered and pledged to employ registered nurses only. Our work for the future will be to obtain some place where we can make arrangements for the disinfection of private nurses on leaving infectious cases, to make some effort to institute a sick-pay insurance—since that is a part of the Pension Fund scheme not open to us—and to make a great effort to place private nursing on a similar basis to that of the London Co-operation. Text-books are to be written specially for the nurses training in the Victoria hospitals by some of the leading members of the medical profession, and one on General Nursing, Hospital Etiquette, etc., by Miss Burleigh, matron of the Melbourne Hospital, and Miss Glover, hon. sec. of the Association.

A meeting of members is to be held each month, and a lecture delivered by some nursing member. Miss Farquharson, matron of the District Hospital, Bendigo, is to be asked to give the inaugural address at the end of January next.

### Christmas Books.

(*Continued from page* 159.)

### BOOKS FOR BOYS.

THE OTHER BOY. By Evelyn Sharp. (Macmillan and Co., Limited. 4s. 6d.)

THIS is a good book for small boys, and girls for that matter too. "The Other Boy" is the nephew of Sir Anthony Marshfield, and is a playmate of the young Irwins, who are not in quite the same circumstances as himself. Tony Marshfield is inclined to be priggish at first but soon finds his equal in Ted Irwin, and the two become fast friends, and despite one or two mishaps the young party seem to enjoy themselves immensely.

THE CRUISE OF THE "KATHERINA." By J. A. Higginson. (Thomas Nelson and Sons. 1s.)

MR. HIGGINSON has written an excellent boys' book, brimful of excitement and adventure; the only fault, if fault it can be called, being its brevity. No one could want a better story than that unfolded in the quest of Charlie Brown and his friend, and their adventures in pursuit of Maggie and her father. A touch of sadness is lent in the tragic death of the devoted native girl Naunee.

THE LOST SQUIRE OF INGLEWOOD. By Dr. Jackson. (Thomas Nelson and Sons. 2s.)

THIS is a book every page of which contains some new adventure. It commences with the flight of Tom Inglewood and his friend Stanley Levers from school, and after a number of thrilling experiences, including the finding of a fortune, and the discovery of Tom's father, who is supposed to have been drowned, the story ends as jerkily as it began. It will be enjoyed by many boy readers, but those of a practical turn of mind will find a difficulty in appreciating the tale.

### BOOKS FOR EVERYONE.

MUSICIANS' WIT, HUMOUR, AND ANECDOTE. By F. J. Crowest. (The Walter Scott Publishing Company, Limited. 3s. 6d.)

THE author having found the demand for a handier edition of his book on Musical Anecdotes sufficient to justify him in bringing out the present volume, offers no apology for so doing. The reader has only to turn to the comprehensive index and pick out at haphazard almost any of his favourite musicians and he will find much that is amusing, a great deal that is instructive, and above all an insight into character which it would, in many cases, be impossible to obtain elsewhere. The illustrations by J. P. Doune are excellent.

LAKE COUNTRY RAMBLES. By W. T. Palmer. (Chatto and Windus. 6s.)

IN an interesting way the picturesque side of our great English Lake Country is depicted in some three hundred pages of reminiscence. The author's aim is a distinctly good one, namely, to convey to his readers the result of his observations in Lake-land at the different seasons of the year. An intimate knowledge of the habits and customs of the inhabitants, and also of the country, is the whole justification for this charming work, a great deal of which is of necessity devoted to sport.

RACQUETS, TENNIS, AND SQUASH. By Eustace Miles (Ward, Lock, and Co. 5s.)

NO one is better qualified to contribute a volume to the Isthmian Library on these three national indoor games than the amateur champion of the world at racquets, tennis, and squash racquets. Mr. Miles quite rightly advocates the adoption of the latter game for all whose working life is spent in our great cities. Lovers of these games will do well to follow the author's many useful hints, and novices cannot find a better introduction to three unrivalled means of taking indoor exercise.

AURORA LEIGH AND OTHER POEMS. By E. M. Browning. (Henry Frowde. 3s. 6d.)

NO one could possibly find a more suitable Christmas present than this beautiful little pocket edition of Elizabeth Browning's celebrated poem, neatly bound in cloth and printed on India paper. These poems form one of the volumes of the "Oxford Miniature Poets."

ANNA OF THE FIVE TOWNS. By Arnold Bennett. (Chatto and Windus. 6s.)

THE scene is laid amongst the Wesleyan Methodists of the north. The author deals with the life of Anna Tellwright and her difficulty in feeling influenced by the doctrines of her religious associates. The character of her miserly old father is well drawn, and his control of Anna's money forms one of the principal features of the story. There is nothing very exciting about the tale, but it is none the worse reading because it deals with a side of religious life but little written of in a novel.

THE PLAGUE OF THE HEART. By Francis Prevost. (Ward Lock and Co, Limited. 6s.)

THE "Siege of Sar" is the first of the three stories which comprise this volume. It is a stirring tale of Indian frontier warfare and concerns the way in which Rose Chantry, whose husband falls soon after the trouble begins, is compelled to join the retreating force from Sar under the command of Captain Terrington. The perils of the retreat both on account of the enemy and of the extreme cold, force these two people into one another's society in a delightfully unconventional way, and the reader is left to draw a natural inference as to the result. "Her Reputation" concerns the machinations of a married lady which are, needless to say, the ruin of her own happiness and the means of spoiling that of Terence, the central figure in this clever piece of writing. "The Measure of a Man" is on similar lines to its predecessor, but points a happier moral in tracing the passions and adventures of Maurice Caragh, a weak specimen of mankind. Taken as a whole the three stories show a keen insight on the author's part into a particular phase of modern life.

WORDSWORTH'S POEMS. (George Newnes, Limited. 1d.)

THIS volume, which begins with the life of the great poet, is a wonderfully well-printed pennyworth. In the same series is issued "Robin Hood's Adventures."

HOW TO BUY A CAMERA. By H. C. Shelley. (George Newnes, Limited. 1s. 6d.)

A WANT will be supplied by "The How to Buy Series," and at the present time when the camera is within reach of all who take the trouble, or have the time, to enjoy the benefits which it confers, this guide to its selection and purchase will indeed be welcome.

ROSALYNDE. By Thomas Lodge. (George Newnes, Limited. 3s.)

THE Caxton Series comprises a selection of famous classics, and amongst them this fine old work appears. The quaint philosophy and charming poetry of Thomas Lodge must appeal to all whose love for books is something higher and more educated than that of the indiscriminate reader.

THE GREEN FLAG AND OTHER STORIES OF SPORT AND WAR. By A. Conan Doyle. (George Newnes, Limited. 6d.)

HERE is a volume which is within reach of every one, and is well worth the modest sixpence which is asked for it. "The Croxley Master" vividly recalls the reader to "Rodney Stone." Each story will be found to be better than the last by those who like light reading of all kinds.

The Cat Manual. By Dick Whittington. (George Newnes, Limited. 1s. 6d.)

This handy little book contains much information which will be instructive and useful to all lovers of cats. The illustrations are very good.

The Lady Killer. By H. de Vere Stacpoole. (T. Fisher Unwin. 6s.)

We cannot imagine anyone reading this book seriously. It is a disjointed tale which deals with the baser side of human nature in a morbid vein, and is not in our opinion sufficiently healthy in tone to justify what other failings it may possess.

The Prisoner in the Dock. By James Greenwood. (Chatto and Windus. 3s. 6d.)

Everyone is aware of the existence of the various police-courts, but the majority of the newspaper reading public has no very definite ideas about them, beyond the fact that they are Courts of Summary Jurisdiction for meting out punishment to criminals. In a thorough yet entertaining style Mr. Greenwood, evidently a keen student of his fellow beings, has written a book on the subject. His anecdotes are excellent, his information is exhaustive, and as far as we know he has touched upon a subject which though within the daily ken of all of us, has not been attempted in book form before. The best chapter in our judgment is "Advice Gratis" in which the author details many instances of the way the magistrates dispense advice to the all and sundry applicants who care to go to him before the business of the Court begins. The remarks and objections raised by Mr. Greenwood to the Inebriates Act, or rather to the impossibility of its provisions being carried into effect will have the sympathy of every right minded reader of "The Prisoner in the Dock."

Fenton's Quest. By M. E. Braddon. (George Newnes, Limited. 6d.)

Though a trifle melodramatic this novel will prove an excellent companion wherewith to while away the time, and is well worth perusal on a Christmas holiday.

Stanhope. By E. L. Haverfield. (Thomas Nelson and Sons. 3s. 6d.)

For a story of peculiar charm and interest we can heartily recommend "Stanhope." It is a novel of the time of the Civil War and a history of two young orphans—Miles Courtney and Lionel Stanhope. Their estates adjoin, but circumstances divide them, Miles fighting for the cause of Parliament, and Lionel joining the Royalist ranks. There is a mystery attaching to Dorothy Stanhope, Lionel's sister, who disappeared soon after her birth. An old Puritan adopted her, however, and all comes right in the end. The description of Hepton Farm and its two sweet inmates, Mara and Hope, is admirably written, whilst the characters of Ishmael and Zechariah are excellent. It will be a hyper-critical reader indeed who cannot appreciate the charm of this delightful little work, which is effectively illustrated by R. Hope.

No Other Way. By Walter Besant. (Chatto and Windus.) 6s.

The author of "All Sorts and Conditions of Men" has left a great legacy to lovers of story-books in his excellent "No Other Way." The history of Isabel is full of real life and charm. Her marriage with the negro Adolphus Truxo, in Newgate Gaol, was the sacrifice she had to pay for her worldly extravagance, but it was a sacrifice dearly bought, for the negro escaped for the time being from the hands of the law, and the creditor did not cease to pester her. Olive Macnamara, the once helpless bankrupt and brilliant advocate, renders her timely assistance on more than one occasion, and the novel closes with his marriage to the daughter of Isabel's drunken creditor. The book is admirably illustrated by Mr. Charles D. Ward.

False Cards. By Hawley Smart. (Ward, Lock and Co. Limited. 6d.)

A good breezy story and like all those which emanate from this author's pen one that will find especial favour at the hands of book-lovers who want something clever and interesting to read in the holidays.

### RELIGIOUS BOOKS.

A Manual for the Sick and Sorrowful. Arranged by E. S. L. (Elliot Stock. 3s. 6d.)

This, as its name implies, is a collection of writings and hymns, and is compiled in three parts. It will be of great use to those whose duties lie amongst human suffering, and will bring consolation and religious peace to many troubled souls.

The Mount of Olives. Edited by L. I. Guiney. (Henry Frowde. 1s.)

Carefully edited, with ample and lucid notes, this revival of Henry Vaughan's work will be welcomed by theological historians and lovers of religious manuals.

### MAGAZINES AND CHRISTMAS NUMBERS.

The Christmas number of "Cassell's Magazine" comprises splendid stories by Ian Maclaren, Egerton Castle, Jerome K. Jerome, and others, besides the opening chapters of what will be a good tale by A. T. Quiller-Couch. Each number contains five Rembrandt photogravures and a splendid large plate of Mr. Julius Price's well-known painting, "Good-bye Sweetheart." "Chums," that deservedly popular boys' paper is well bound and full of good serials and short tales. Quite apart from the excellent athletic matter it contains, there are a large number of beautiful coloured plates, which combine to make the volume an excellent Christmas present for all sorts and conditions of boys. The collection of last year's "Cassell's Magazine" is a fund of literary and art merit. "A Gentleman of Devon" is one of the many serial stories it contains, and through its pages Mr. D. H. Parry, with the aid of Mr. Edward Reade's illustrations, traces the history of Sir Walter Shilingford, who served at the time of the Spanish Armada. There are innumerable illustrations, several excellent photogravures, all aiding to make the book as instructive and interesting as usual. "Pears' Annual," is as hardy as ever, and this Christmas is issued with three coloured plates, "The Ferry," from the water colour drawing by W. S. Coleman; "Spring Blossoms," by the same artist; "A Breezy Day in the Mediterranean," after Birkett Foster, R.W.S.; and "Impudent Hussies," from the original painting by Miss Mary Groves.

The "Windsor Magazine" contains contributions by S. R. Crockett, Sir Henry Irving, Rudyard Kipling, Rider Haggard, and many other well-known people. It contains over 200 pages and is full of interest and excellent illustrations.

Little Folks. (Cassell and Co., Limited.)

This is the recognised children's magazine, and certainly should occupy as prominent a place as ever amongst their list of Christmas gift books this season. The illustrations throughout are as good as ever, and six coloured plates add considerably to the effect of a very pleasing volume.

Tiny Tots: a Magazine for very Little Folks. (Cassell and Co., Limited.)

This is, as its name betokens, the annual consort of "Little Folks," and though on a slightly smaller scale, it contains many typical stories and rhymes, and is very suitably illustrated.

### The Nurses of the Hospital for Sick Children.

INTERVIEW WITH THE MATRON. BY OUR COMMISSIONER.

No London hospital excites keener interest at the Christmas season than the well-known institution in Great Ormond Street, which always attracts so many visitors intent on contributing to the happiness of the inmates. It is therefore a specially appropriate time to show what provision is made for the training and advantage of the large nursing staff on whose capacity and character the little patients depend even more than sick and disabled adults in general hospitals.

Miss Gertrude Payne, the matron of the Hospital for Sick Children, first enabled me to refresh my memory of the Nurses' Home, which was opened nearly four years ago, and thus to realise the substantial improvements which have been carried out for the comfort and convenience of her department.

### The Advantages of the Home.

The contrast before these improvements were made cannot be more forcibly stated than in the matron's own words.

"Before we had this home," said Miss Payne, "the nurses slept in cubicles, which were very low and decidedly unhealthy. Of course, the reception room, the library, and the waiting room for the friends of the nurses, which we now possess, are very much appreciated. But the bedrooms and the bath-rooms were absolutely indispensable. In former times the nurses suffered a great deal from bad sore throats and other serious ailments, owing to the manner in which they were housed. Since the home was opened, we have had very few cases of sickness among them, and only one of typhoid in four years among a staff of about eighty. Also, it is easier to obtain nurses than it was."

### The New Staff Nurses.

"That I can well imagine. What is the number of the existing staff?"

"It is made up of 70 non-paying probationers, 16 paying probationers, 10 ward sisters, the assistant matron, the housekeeper, who is also a trained nurse, and myself."

"You have been matron for some five years, I think?"

"Since June 1897. Previous to that I was home sister for two years and a half."

"You mentioned 70 non-paying probationers. Are not some of these staff nurses?"

"Yes, twelve, all of whom are in their fourth year."

"But your period of training is three years?"

"Certainly, and the certificate is given at the end of three years, signed by the chairman of the House Committee, the senior physician, surgeon, and matron, to the effect that the probationer has received three years' training in nursing medical and surgical cases, and is competent to undertake the nursing of sick children. But the probationer, having gained her certificate, has the option of staying on as staff nurse at a salary of £30, and during that year she is exempt from night duty. This arrangement has been made since I became matron."

"Is night duty not liked, then?"

"The nurses prefer not to have night duty, which as you can understand, is sometimes more wearing in a children's, than in an adult, hospital."

### Paying and Non-Paying Probationers.

"I should like to know the precise difference between the conditions for non-paying and paying probationers."

"They can very soon be told. The paying probationer who pays a fee of 52 guineas, by four quarterly instalments, comes on trial for three months; the non-paying probationer for two months. The paying probationer has a bedroom to herself; the non-paying probationer has to share her room with another for the first year. This, however, is only because we have still not sufficient accommodation. I hope that in a year or two every probationer will have a separate bedroom. The paying probationer has to defray the cost of her laundry expenses and of her uniform, which are provided free to the non-paying probationer. In other respects there is practically no difference."

"I conclude that at the end of a year the paying probationer may proceed with her training?"

"Yes, and, as a matter of fact, many paying probationers are transferred to the regular staff, though some only wish to stay for one year. Socially, all probationers belong to the same class. At any rate, there are no class distinctions here."

### Age and Hours of Duty.

"What about age?"

"There is a slight distinction. All probationers must be not less than 21, but we receive paying probationers up to the age of 35, while the non-paying must not be more than 30. It is essential for every candidate to furnish evidence of successful vaccination since reaching the age of 15."

"Are there any special features in the regulations for probationers?"

"We offer payment from the outset:—£8 for the first year, £12 for the second, and £18 for the third. As to training, each probationer has at least two months' experience in the diphtheria ward. Lectures are given by the medical officers in elementary anatomy, physiology, and medical and surgical nursing; instruction by the matron, home sister, and ward sisters in the management and nursing of sick children, and in the cooking and preparation of their food. Examinations are held from time to time, and the competence and conduct of each probationer is reported on to me every two months."

"Are the hours on duty in any way exceptional?"

"Only the probationers in their third year are on duty during the night. The hours of duty during the day are from 7 A.M. to 8 P.M. Each probationer is off duty three hours. She is also entitled to three weeks' holiday during the year. The hours of the staff nurses, their time off-duty, and holidays are similar.

### Sisters and Night Duty.

"I presume that the age of eligibility for sisters fits in with the regulations for probationers?"

"Yes, a nurse may become a ward sister at 25, if she has had three years' medical and surgical training here or in another hospital. Our sisters have more time for off-duty than is usual, because, as I intimated in respect to the question of night duty, the work is peculiarly trying. I refer particularly to the unavoidable crying of the children in pain. Sisters breakfast at 8, are on duty at 8.30 and off at 10 P.M. They have always four hours off in the afternoon or evening."

"What about night duty?"

"There is only one sister on night duty, and she undertakes it continuously for twelve months or longer. She is paid at a higher rate than the other sisters. They receive £35 the first year, and £40 the second. She is paid £40 the first year. All the sisters have a month's holiday."

### The Private Staff.

"You have also a considerable private staff?"

"Twenty-five. Most of these, like most of the sisters,

### THE NURSES OF THE HOSPITAL FOR SICK CHILDREN—*Continued.*

were trained at this hospital, and all have had at least three years' training in a hospital of not less than 60 beds. Some of those who were trained here have also had a year's general training."

"Are they under the control of the matron?"

"Under the immediate control of the staff sister, but subject to my general control. The private nurses are provided with indoor and outdoor uniforms, which they are required to wear during the whole time of attendance on a case. When they are not in attendance on a case the nurses reside in the home for the private staff. The salary is £30 for the first and second years, and £35 for the third, with an allowance for washing and a percentage on the sums received by the hospital from the patients nursed by her, varying from 10 to 30 per cent."

### The Nursing of Children.

"How far do you find, in practice, that the training at the Children's Hospital qualifies for other hospitals?"

"My opinion is that if a woman can nurse children she can nurse adult patients. Moreover, we have cases of all sorts in the medical and surgical wards, except fractures. But, as the ordinary age of admission to a general hospital is 24, while ours is 21, many of the nurses who have been trained here for three years then take up general training for three years, while others go to the provincial hospitals which receive them for one year."

"Have you any lack of applications for probationerships?"

"Our average number of applications to enter as non-paying probationers is six a day."

"There is an impression in some quarters that your patients are not always from the class most in need."

"That is a mistake. We do not receive patients whose parents are earning more than 40s. a week, and though we have some quite refined children, many belong to the very poorest parents. I dare say that because all our children are dressed so nicely people run away with the idea that their parents are not poor. But the dresses belong to the hospital."

### The Christmas Entertainments.

"Christmas is, of course, a great time for the children?"

"We do all we can to make them happy. One of the doctors makes up as 'Father Christmas,' and along with one of the sisters puts well-filled stockings in the wards. The nurses sing carols, and there is a Christmas tree and toys for the children. The whole of the staff remain on duty during Christmas Day, for the sole purpose of amusing the children. Then there are out-patients to provide for."

"How many?"

"Several hundreds. The 'Out-patient' department has become so extensive that there is a sister always in charge, with four staff nurses and three probationers to assist her. It requires a lot of management. At Christmas 250 of the poorest of the out-patient children have a tea, a Christmas tree, a 'Punch and Judy,' and a parcel with a garment in it. 'Father Christmas' also gives them each a toy to take home. About the middle of January the entertainments are finished by the nurses going to a theatre or other place of amusement. They go where they please one evening and return to the hospital to a supper."

"You have had a busy time this year?"

"We are always busy, but the bazaar involved a lot of extra work. The nurses' stall realised £521, almost as much as any, and you can imagine that this amount meant a great deal of labour. As to the future, we have now a fully-qualified staff nurse in charge of the operating theatre, along with a probationer. This I hope to make a sister's appointment."

### The Value of Practical Knowledge.

"Do you care to add any general remarks?"

"Only to emphasise the value of the very best training for the nursing of children. It is impossible to exaggerate the importance of practical knowledge as to their management and feeding. Surely, it is an immense advantage for any woman to learn to understand how to treat the ailments of babies. That is why we think the one year's training must be beneficial to young ladies who have no intention of being nurses."

### Everybody's Opinion.

**[Correspondence on all subjects is invited, but we cannot in any way be responsible for the opinions expressed by our correspondents. No communication can be entertained if the name and address of the correspondent are not given as a guarantee of good faith, but not necessarily for publication. All correspondents should write on one side of the paper only.]**

### THE MIDWIVES' INSTITUTE.

"Miss Maule," writing from 12 Buckingham Street, Strand, in reference to an answer given to "R. D." in our "Notes and Queries" column of December 6th, asks us to state that the Society for promoting the Compulsory Registration of Midwives never had any connection with the Midwives' Institute, 12 Buckingham Street, and that the maternity lectures are given at the Midwives' Institute, not at the Nurses' Club.

### CORONATION NATIONAL FUND FOR NURSES IN IRELAND.

"A Dublin Nurse" writes: I observe from your issue of 6 h inst. that "Another Dublin Nurse" writes to you on this subject, with reference to my letter which you were good enough to publish and comment on in the pages of The Hospital of 22nd ultimo. I am not on sufficient terms of intimacy to enable me to say whether you are "kind-hearted" or hard-hearted, or have any heart at all! this is not a case of cardiac affection! But my sister nurse's letter reads as all but an apology for the proposed Irish Fund; and I venture to think that it is both contradictory and inaccurate. At the commencement she states that "there is no intention to start a Separatist Fund," but, towards the end, she writes that "the feeling is growing amongst nurses in Dublin that the time has come when they should form some definite organisation amongst themselves!" I am not a nurse of yesterday, and have served my time in England with the rest, but I know of no such general feeling in Dublin. All success to a benevolent fund for the purpose of making charitable grants to distressed nurses, or to one that would aid our Irish nurses to become members of the Royal National Pension Fund; but as you, Mr. Editor, have advised, let not a farthing of our earnings go to any purely pension fund on this side of the Channel. We nurses should recognise a distinction. There may be a purely charitable fund for those who may come to be in distress (let us hope, not the 3,860!), but there is also the non-charitable return for our own industry and thrift, in the shape of the Royal National Pension Fund policy. Even well-meaning matrons and medical gentlemen in Dublin are not likely to succeed in convincing many nurses here that the premium of the English Fund is such as to be beyond the reach of most of those nurses I refer to, or that an Irish Fund could ever confer equal benefits and financial safety for less money. Whatever may be the result in this immediate matter, your having opened your pages to a brief correspondence of the kind —I will not seek room again—cannot but be beneficial to our self-sacrificing profession, in leading them, perhaps, more often to reflect on the time "when night cometh and no man can work."

### Appointments.

[No charge is made for announcements under this head, and we are always glad to receive, and publish, appointments. But it is essential that in all cases the school of training should be given.]

Ashton-under-Lyne Borough Hospital.—Miss Sara W. McKillop has been appointed charge nurse. She was trained at Belvidere Hospital, Glasgow, and has since been head nurse at Cleckheaton Hospital, Yorkshire, and charge nurse at Southport Borough Hospital. She has also done private nursing.

Barnet Union.—Miss Florence Gray has been appointed assistant nurse. She was trained by the Meath Workhouse Nursing Association at St. Peter's Home, Kilburn.

Battle Workhouse. — Nurse Alice Reed has been appointed assistant nurse. She was trained by the Meath Workhouse Nursing Association at St. Luke's Hospital, Halifax, and has been assistant nurse at Plymouth Infirmary.

Borough Isolation Hospital, Plymouth.—Miss Helena K. Repton has been appointed night sister. She was trained at Leeds General Infirmary.

Brecknock County and Borough Infirmary. — Miss Florence M. Atkins has been appointed nurse-matron. She was trained at Bristol Royal Infirmary, where she has since been sister. She has also been attached to a nursing home at Fulham.

Chelsea Hospital for Women.—Miss E. M. Edwards has been appointed matron. She was trained for four years at St. Bartholomew's Hospital, London, and has since been night sister and assistant matron at Chelsea Hospital for Women for 14 months, and matron of Ilkley Hospital for three years.

City Hospital East, Liverpool.—Miss Margaret Park Cross and Miss Martha Reeve have been appointed charge nurses. Miss Cross was trained at Belfast Royal Infirmary; Miss Reeve was trained at Brownlow Hill Infirmary, Liverpool, where she has since been charge nurse.

City Sanatorium, Peterborough.—Miss Florence Firley has been appointed charge nurse. She was trained at the City Fever Hospital, Wakefield, and the City Sanatorium, Peterborough.

Cuckfield Union Infirmary.—Miss Amy Louisa Burgess has been appointed superintendent nurse. She was trained at Woolwich Workhouse Infirmary, where she has since been sister. She has also been temporary nurse in Sutton School. She holds the L.O.S. certificate.

Fairford Cottage Hospital.—Miss Alice T. Brierley, L.O.S., has been appointed matron. She was trained at Guy's Hospital, London, and the Greek Hospital, Alexandria, and has since been matron of the Tetbury Cottage Hospital, and the West Highland Cottage Hospital, Oban.

Folkestone Sanatorium. — Miss Clara Alice Barling has been appointed matron. She was trained at St. Mary's Hospital, Paddington, and held temporary post as sister of the operating theatre for several months, and holiday sister in the medical and surgical wards. Since January she has been sister of the women's and children's ward at the Victoria Hospital, Folkestone. She is a certificated masseuse.

Fulham Infirmary, Hammersmith.—Miss Janette Haley and Miss Annie Latchford have been appointed sisters. Miss Haley was trained at Whitechapel Union Infirmary and has since been sister at Cleveland Street Sick Asylum, London, and district nurse. Miss Latchford was trained at Nottingham Workhouse Infirmary, and has since been temporary charge nurse at Leeds Workhouse Infirmary and staff nurse at Islington Workhouse Infirmary.

Houghall Isolation Hospital.—Mrs. John Golder has been appointed superintendent nurse. She was trained at Belvidere Hospital, Glasgow, and has since held the post of sister at the Sanatorium, Hull. She has also done two years' private nursing and district work, both in Yorkshire and Lincolnshire, and has held the post of senior charge nurse at Knightswood Hospital, Partick, Glasgow.

Ilkeston Hospital.—Miss Eva Goring has been appointed staff nurse. She was trained at Lowestoft Hospital, and has been staff nurse at Northwich Victoria Infirmary, Cheshire.

Infectious Diseases Hospital, Brandon. — Miss Madeline Aspinwall has been appointed nurse in charge. She was trained at St. Mary's Hospital, Paddington, and has since been charge nurse at the South-Eastern Fever Hospital, London, sister at the City Fever Hospital, Sheffield, and sister at Fylde Hospital, Lytham.

Milton Hospital, Portsmouth.—Miss Emily Taggart has been appointed night sister. She was trained at Leeds Union Infirmary, and has since been ward sister at Monsall Fever Hospital, Manchester.

National Hospital, Queen's Square, Bloomsbury.—Miss M. G. Tottenham has been appointed ward sister. She was trained at the Royal Hants County Hospital, Winchester, where she held the post of night sister and surgical ward sister, and has since been a Queen's nurse at Leeds, and charge nurse at the Brook Fever Hospital, Woolwich, London. She holds the L.O.S. certificate.

Southwark Infirmary.—Miss Margaret Palmer has been appointed sister. She was trained at Guy's Hospital, London.

Union Infirmary, Newtownards, County Down.—Miss Letty Stewart has been appointed night nurse. She was trained in the Union Infirmary and Fever Hospital, Belfast.

Wolverhampton General Hospital.—Miss Katharine Frances Cooper has been appointed theatre and home sister. She was trained at Northampton General Infirmary, and has since been sister of women's surgical wards in the same institution, and sister of men's surgical wards in the Metropolitan Hospital, London.

### "The Hospital" Convalescent Fund.

The Honorary Secretary begs to acknowledge with thanks the receipt of 5s. from "Chigwell."—The contribution from Sister Elizabeth acknowledged in our last week's issue was for 5s., and not 10s. as therein stated.

### TRAVEL NOTES AND QUERIES.

Spain in March (Espagne).—I am pleased to help you, but you must write more fully and tell me exactly what money you can afford each. To say that you want to spare expense is too vague. Spain is a dear place to travel in, and cheap pensions are unknown, except in such places as Malaga. Seville is so far south that your journey would be very costly, do it how you will. I do not think the line you mention would be any advantage when all supplementary costs were paid, but write me what you can each spend and I will find out the cheapest way for you. Are you good travellers and able to put up with long runs, because the more breaks there are in your journey the more costly it is? The fare, second return *viâ* Newhaven and Paris to Madrid, is about £9. St. Sebastian about 30s. less. Northern Spain is very cold in March, and I am doubtful if you could sketch out of doors at that season, the temperature is much below that of South France. If you want to sketch and climb why not go to San Jean de Luz under La Rhune and the "Trois Couronnes." From there I used to cross the frontier to San Sebastian and Fontarabia frequently. I will tell you more when I have heard again. Some articles are about to be published concerning that corner of the world.

### Echoes from the Outside World.

### Movements of Royalty.

THE King and Queen, who were accompanied by Princess Victoria, terminated their visit to the Earl and Countess Howe on Monday, after a stay of five days. The King spent much of his time in shooting, the ladies of the party generally joining the gentlemen at lunch. On the Friday, the Gopsall children assembled at the wayside and sang the National Anthem as the King passed along, and later in the day were fortunate enough also to see the Queen and Princess Victoria. Saturday was mostly devoted to golf, but the King planted an oak tree in commemoration of his visit, and also inspected the prize cattle at the stock farm belonging to his host. M. Casano's orchestra performed every evening in the picture gallery, and the house party included the Duke of Devonshire and Mr. Balfour.

On Sunday the King and Queen attended Divine service at Gopsall, the musical portion being rendered by the Lichfield Choir. The Queen afterwards spoke to three of the young choristers, complimenting them upon the possession of such charming voices, and handing to each a leather writing-case bearing the recipient's name "Gopsall Park, December 14, 1902." The King presented the organist, Mr. Perkins, with a diamond pin with the monogram E.R.

In order that a number of people should have an opportunity of seeing their majesties on Monday they had consented to drive to Shackerstone Station in open carriage, and loyal subjects assembled in thousands long before 11 o'clock, the hour for departure. The journey to Euston was performed in the new Royal train without a stop. On Tuesday the King attended the christening of the infant son of Lord and Lady Castlereagh and stood sponsor.

### The Queen's Christmas Dinner.

QUEEN ALEXANDRA, knowing well that, owing to the war in South Africa, there will be many sad homes this Christmas where the loss of the breadwinner will cause not only sorrow of mind, but often a distressing failure of the physical comforts with which the glad season is associated, has signified her intention of giving a Christmas dinner on Saturday, the 27th inst., at 2 P.M., to the widows and children living in London and the suburbs of all the soldiers and sailors of the Imperial and Colonial forces who lost their lives during the campaign or who have died from the effects of service in South Africa since their return home. Her Majesty desires, "in this the Coronation year, to express her heartfelt sympathy with those who have suffered the cruel bereavement of war, and to wish that happiness and comfort may, with God's blessing, be theirs in the coming year." As a further proof of the thoughtful and practical nature of her generosity it has been arranged that the expenses of the guests going to and from the dinner shall be defrayed by the Queen. The festivity is to be held at the Alexandra Trust, City Road.

### The Queen's Birthday Cake.

The kindly thought of Queen Alexandra for the child-actors in "Quality Street" caused great excitement at the Vaudeville Theatre on Friday evening. A large birthday cake had been made at Sandringham in honour of her Majesty's birthday. It was quite a work of art, in two tiers, beautifully ornamented with flowers made out of sugar, and of such an enormous size that it took two men to carry it. This the Queen had forwarded from her Norfolk home to the theatre in the Strand, with the express wish that it should be cut up and given to all the children who appeared before her Majesty at Windsor Castle. A party was given after the performance, when the cake was cut and the health of the donor drunk with much enthusiasm by the delighted youngsters.

### The Trouble with Venezuela.

OWING to the evasive attitude of Señor Castro, the President of Venezuela, the British and German Governments last week sent in a final ultimatum. The British note pressed for satisfaction of claims of compensation for injuries suffered by British subjects and damage to their property in the last revolution; the German note demanded payment of interest on the German loan, and also for compensation to German subjects. No notice being taken of either of these notes, for a period of 48 hours, the British and German warships seized the Venezuelan fleet of four vessels lying in the harbour of La Guayra. This was accomplished by ten German and four British cutters, who went alongside the Venezuelan vessels, ordered them to surrender, and without a shot being fired, seized them in the names of the German Emperor and the King of England. Three were afterwards taken out of harbour and sunk at two o'clock in the morning, because they were stated to be unseaworthy. The fourth was spared, and now flies the German ensign. Most of the English and German residents in La Guayra were rescued and taken on board the English or German ships, and President Castro summoned the Venezuelans to arms. The populace fortified the city, the women helping by filling the sandbags of which impromptu ramparts are being made. On Saturday Puerto Cabello was bombarded for 45 minutes, and two people were wounded. On Monday Lord Lansdowne announced in the House of Lords that a proposal had been received, through the United States Government, from the President of Venezuela, that the dispute should be referred to arbitration. On Tuesday he said that it was intended to establish a blockade of the Venezuelan ports, but not land a British force.

### The League of Mercy.

ONE of the most interesting social events of last week was the dance on Wednesday night at the Empress Rooms, Kensington, under the patronage of the Prince and Princess of Wales. The dance was arranged by the Lady Presidents of the Western Metropolitan District of the League of Mercy with a view to make the objects of the League better known, and in the hope of enlisting new workers. The fine ballroom was beautifully decorated with palms and flowers, kindly supplied by Mrs. Leopold de Rothschild, and the magnificent private band which the Right Hon. Sir Ernest Cassel, K.C.M.G., generously placed at the disposal of the Lady Presidents was a great attraction. Since the establishment of the League, of which the King is patron, as well as Sovereign of the Order, by Royal Charter in 1899, £14,000 has through its efforts been handed over to King Edward's Hospital Fund.

### The New Othello.

THOSE who were delighted with Mr. Forbes Robertson's "Hamlet" will probably be equally pleased with his recent production. "Othello," as seen at the Lyric, is a stirring performance, the everyday story of an everyday crime clothed in the beautiful language of a great dramatist. Mr. Robertson, as the hero, has not feared to strike out for himself original lines, refusing to be hampered by "tradition" in any form, and he is always thoroughly natural. He is a gaunt-looking rather than a "lusty" Moor in the first acts, but always with a noble air, playing quietly and gently. In the later acts, touched by the frenzy of jealousy, he is almost beside himself with passion, and carries all before him, including his audience. Miss Gertrude Elliott as Desdemona is sweet and refined—a striking contrast to Miss Lena Ashwell's Emilia, a vigorous performance, touched with many lights and shades. Mr. Herbert Waring as Iago is villainous, but not subtlely villainous; Mr. Ben Webster as Cassio thoroughly realises all that is required of him and the whole performance—well staged and well mounted—is one of more than ordinary interest.

### Notes and Queries.

**The Editor is always willing to answer in this column, without any fee, all reasonable questions, as soon as possible.**

**But the following rules must be carefully observed:—**

**1. Every communication must be accompanied by the name and address of the writer.**

**2. The question must always bear upon nursing, directly or indirectly.**

**If an answer is required by letter a fee of half-a-crown must be enclosed with the note containing the inquiry, and we cannot undertake to forward letters addressed to correspondents making inquiries. It is therefore requested that our readers will not enclose either a stamp or a stamped envelope.**

*Dispensing.*

(92) Will you tell me where I can be taught dispensing? Is there a hospital or institution where I could give my services in house work and a small premium in return for instruction, as I cannot afford to pay fees?—*A. B. S.*

No public institution would receive you on the terms you mention, but perhaps you could find a provincial chemist who would take you as a pupil for three years on reciprocal terms. In this case you should write to the Secretary of the Pharmaceutical Society, 17 Bloomsbury Square, W.C., and ascertain if your prospective employer is properly qualified.

*Maternity.*

(93) 1. Will you kindly tell me the easiest means of qualifying as midwife? 2. Is the L.O.S. the only qualification taken? I am a district nurse and my salary is not large.—*L. R. J.*

1. The best and most satisfactory way is of course to go to a lying-in hospital for the three months' course of training. The Secretary of the Midwives' Institute, 12 Buckingham Street, Strand, would be able to advise you as to the nearest and cheapest to your place of residence. You might also write to the Secretary of the London Obstetrical Society, 20 Hanover Square, London, W., and ask for syllabus of the examination, and arrange with one of your local medical men to prepare you for it. 2. It is the only English qualification absolutely necessary for maternity nursing.

*A Matron's Right.*

(94) *G. W.* is anxious to know if a matron has a right to prevent her nurses visiting friends when off duty, and also to forbid a nurse corresponding with friends whilst at a non-infectious case?

A matron has no right to interfere with the private affairs of the nurses in her employ. She could only claim, therefore, to object to a nurse's visiting her friends in her off-duty hours if that nurse were, by so doing, omitting to take the proper exercise for which such time is allowed.

*Regimental Nurse.*

(95) Please tell me if any regiment has a nurse attached to it on the same lines as those of a district nurse, and, if so, what is the best way of securing such an appointment?—*A. I. P.*

Army nursing is entirely under the control of the matron-in-chief, Queen Alexandra's Imperial Nursing Service, Horse Guards, S.W., to whom all applications must be made. There are, however, no nurses attached to regiments.

*Sharing Cottage.*

(96) A lady obliged to give up nursing on account of a bad illness from overwork, would like to find another to share her small cottage with her. Is there any institution to which she could apply for information?—*K. M.*

Write to the Secretary of the Royal British Nurses' Association, 10 Orchard Street, W. The Association is endeavouring to aid its aged and disabled members by finding, and helping to provide, homes for them.

**Standard Books of Reference.**

"The Nursing Profession: How and Where to Train." 2s. net; post free 2s. 4d.

"Burdett's Official Nursing Directory." 3s. net; post free, 3s. 4d.

"Burdett's Hospitals and Charities." 5s.

"Hospital Expenditure: The Commissariat." 2s. 6d.

"The Nurses' Dictionary of Medical Terms." Cloth, 2s.; leather, 2s. 6d. net.; post free, 2s. 8d.

"Burdett's Series of Nursing Text-Books." 1s. each.

"A Handbook for Nurses." (Illustrated). 5s.

"The Physiological Feeding of Infants." 1s.

"The Physiological Nursery Chart." 1s.; post free, 1s. 3d.

All these are published by the Scientific Press, Ltd., and may be obtained through any bookseller or direct from the publishers, 28 and 29 Southampton Street, London, W.C.

### For Reading to the Sick.

CHRISTMAS-TIDE.

Lo, while the woods are wrapped in mystic guise,
And every frosted tree, and tower are traced
With magic lines—with wonder—while effaced
Are all the scars that lay before the eyes,
A wondrous time draws near! Love's old surprise
Again is new; and every home is fain
To greet some occupant with heart, again—
Such loveliness in ancient custom lies!

Hark, to the bells of yonder church! The mirth
Of playful laughter, and the tripping feet;
The welcomings that, on the air, are sweet;
As if the reign of Peace had come to earth!
Goodwill is born; it is the Christmastide,
Which tells His birth who for our healing died!

*W. J. Gallagher.*

The love of God in Christ Jesus, the love of God in His Presence among us, in his acceptance of the extremities of our lot, in His infinite compassion and infinite patience, in the tenderness of His inexhaustible sympathy—the love of God in the manger of Bethlehem, the love of God in the awful Passion at Jerusalem, in Jesus Christ dying for the world—that was the "glad tidings" which fills the New Testament.—*R. W. Church.*

"The era of *humanity* is the era of the Incarnation." The influence of Jesus Christ has radiated from His own Person into the outer world. It is to Christianity that slavery owes its suppression. It is through Christianity that the rights of the poor are more and more recognised and respected. It is to Christianity that the sick and suffering owe the blessings of loving care in our hospitals. It is by Christianity that woman has been raised from degradation to honour. It is through Christianity that the nations are being taught to regard each other as brethren.—*V. Staley.*

Blessed Jesus, who hast left us a perfect pattern of self-denial and self-forgetfulness, help us to offer ourselves, our souls and bodies, a sacrifice holy, acceptable unto Thee.

*H. J. Wilmot Buxton.*

Yea, Christ Himself
Is with us; lo! the Shepherd-King of the Church
Abideth in the fields, and watcheth o'er
His flock by night. But who shall give Him praise
For this sweet service? Who shall celebrate
The name of God by night?
Whilst we are sleeping, those to whom the King
Has measured out a cup of sorrow, sweet
With His dear love, yet very hard to drink,
Are waking in His temple. Sweet low songs,
Broken by patient tears, arise to God:
They lift their hands and bless God in the night.

